# Pericardial Effusion during Proton Therapy in a Patient with Chemorefractory Hodgkin Lymphoma

**DOI:** 10.14338/IJPT-21-00010

**Published:** 2021-10-18

**Authors:** Ashley Way, Savas Ozdemir, Barbara Berges, Nataliya Getman, Xiaoying Liang, Nancy P. Mendenhall, Graham Collins, David Cutter, Raymond B. Mailhot Vega

**Affiliations:** 1 Department of Radiation Oncology, University of Florida College of Medicine, Jacksonville, FL, USA; 2 Department of Radiology, Division of Nuclear Medicine and Molecular Imaging, University of Florida College of Medicine, Jacksonville, FL, USA; 3 Oxford Cancer and Haematology Centre, Oxford University Hospitals NHS Foundation Trust, Oxford, UK; 4 Department of Oncology, Oxford University Hospitals NHS Foundation Trust, Oxford, UK

**Keywords:** Hodgkin lymphoma, particle therapy, proton therapy, pericardial effusion, radiotherapy

## Abstract

We present a case of recurrent pericardial effusion presenting during proton therapy in a 24-year-old female receiving mediastinal treatment for classical Hodgkin lymphoma. Pericardial effusion is typically considered an event accompanying lymphoma diagnosis or as a subacute or late effect of radiotherapy. Rarely has it been described as occurring during radiation treatment with photon-based radiotherapy, let alone proton therapy. It is unclear what underlying cause triggered recurrent effusion in this patient. Identifying and managing pericardial effusion during treatment delivery is important to consider as it may affect radiation dosimetry, particularly with proton therapy. Doing so will help ensure patients receive optimal treatment and minimize the risks of morbidity and mortality.

## Introduction

In the United States, 10% of all new lymphoma cases are diagnosed as Hodgkin lymphoma (HL), representing 0.5% of total cancer diagnoses. Symptoms are often nonspecific including nontender supradiaphragmatic lymphadenopathy, pruritus, high fevers, night sweats, and/or weight loss [1]. Classic HL is a curable disease with the use of chemotherapy and, in selected cases, consolidative radiotherapy (RT). With treatment, HL patients have, on average, an excellent prognosis with a relative 5-year survival of 85% across all stages of diagnoses [2, 3]. HL treatment strategies are determined by lymphoma stage, response to chemotherapy—often assessed by fluorodeoxyglucose (FDG)-18 positron emission tomography/computed tomography (PET/CT)—and the need for or access to adjuvant RT [4]. Unfortunately, treatment itself has been associated with unwanted side effects, including radiation-related pericardial effusion (PCE) which, if it occurs, most often presents a year after the final RT treatment [5].

The appearance of recurrent nonmalignant PCE in an HL patient during concurrent radiotherapy with double-scatter proton therapy is unexpected and has not been previously reported in the peer-reviewed literature, warranting further investigation. Our goal is to discuss recurrent nonmalignant PCE and formulate a suggested strategy for the timing and nature of appropriate investigation and intervention. Understanding the pattern of recurrence, PCE treatment, and patient response to surgical intervention are important factors to consider during initial patient planning.

### Case

**Figure 1.**
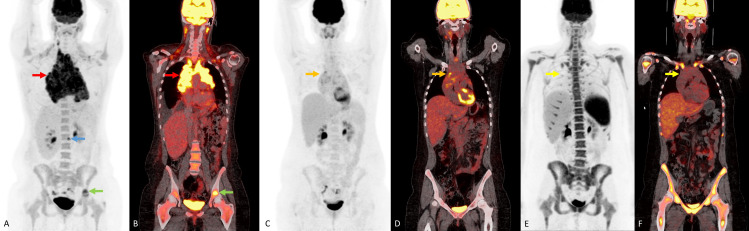
FDG-18 PET/CT MIP image (A) and coronal FDG PET/CT fusion images (B) for initial staging demonstrate FDG-avid bulky mediastinal and cervical nodes (red arrow). FDG-avid osseous lesions at the L2 vertebral body (blue arrow) and FDG-avid osseous lesion in left acetabulum (green arrow). Also note the physiologic brown fat uptake in the cervical and supraclavicular regions bilaterally. F-18-FDG PET/CT MIP (C) and coronal FDG PET/CT fusion image (D) for restaging after 2 cycles demonstrate decreased sized and FDG activity in the mediastinal and cervical nodes (orange arrow) consistent with a Deauville 4 response. F-18-FDG PET/CT MIP (E) and coronal FDG PET/CT fusion image (F) after chemotherapy escalation demonstrate a further decrease in FDG uptake in the mediastinal and cervical nodes (yellow arrow) consistent with a Deauville 2 response. Also note the diffuse increase uptake in bone marrow and spleen, likely secondary to chemotherapy. Abbreviations: FDG, fluorodeoxyglucose; MIP, maximum intensity projection; PET/CT, positron emission tomography–computed tomography.

**Figure 2.**
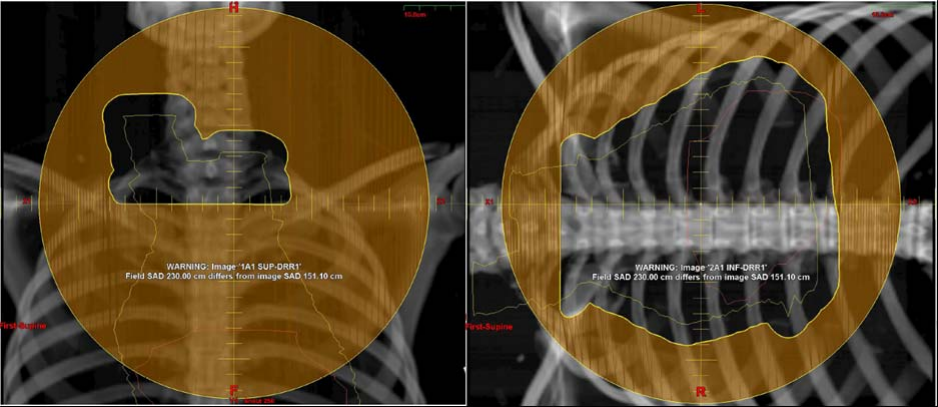
(A) Fields for treatment are shown in the figure. The left panel demonstrates field 1A1 and the right panel demonstrates field 2A1. The yellow contour demonstrates the internal target volumes, and the orange contour is the heart.

A 25-year-old white woman presented to an outside institution reporting several months of night sweats and itching with progressive facial swelling, trouble swallowing, and respiratory distress. Further evaluation led to the discovery of PCE, which was subsequently drained and evaluated, revealing no evidence of malignancy on cytology. Two months later, CT of the anterior/posterior chest revealed a large soft tissue mass in the mediastinum measuring 22 cm in greatest dimension, compressing the superior vena cava and trachea with demonstration of recurrent PCE. This second PCE was treated with pericardiocentesis and again confirmed as a nonmalignant effusion. A biopsy procedure and workup confirmed classic HL, stage IVB, with osseous disease but lymphadenopathy confined to mediastinum and neck. She received doxorubicin, bleomycin, vinblastine, and dacarbazine ×2 with a Deauville 4 response in the mediastinal bulk disease, prompting chemotherapy escalation to eBEACOPDac (escalated bleomycin cyclophosphamide dacarbazine doxorubicin etoposide prednisolone vincristine). After 3 cycles, PET/CT noted a Deauville 2 response in the mediastinal mass (**Figure 1**). She was referred for proton therapy for consolidation of the bulk disease and prescribed 30.6 GyRBE of involved-site RT to the slow-responding bulky mediastinal lesion. She underwent simulation 8 days after finishing her last cycle of eBEACOPDac (8 days after day 21), and radiotherapy delivery began 13 days after simulation. Radiotherapy delivery consisted of 2 plans based on junction, both consisting of 2 anterior fields as follows: an A set with an anterior field at 0° and a second with a couch kick of 90° and similar angle, with a B set approximating the A set. The A set is represented in **Figure 2** in addition to the internal target volumes and heart contours.

**Figure 3.**
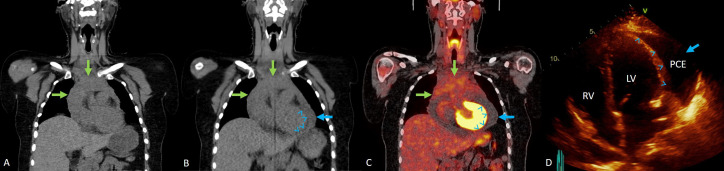
Coronal CT scan for proton therapy planning (A) shows residual enlarged mediastinal lymph nodes (green arrows). No pericardial effusion is noted. Coronal CT proton therapy fraction 8 (B) shows residual enlarged mediastinal lymph nodes (green arrows), and a new pericardial effusion (blue arrow heads and blue arrow). FDG-18 PET-CT fusion image after proton therapy fraction 8 (C) demonstrate FDG uptake in mediastinal lymph nodes (green arrows) consistent with a Deauville 3 response. No FDG uptake in pericardia effusion is demonstrated (blue arrow heads and blue arrow). Echocardiogram (D) confirms pericardial effusion (blue arrow heads and blue arrow) prompting patient intervention with pericardial window. Abbreviations: CT, computed tomography; FDG, fluorodeoxyglucose; PET, positron emission tomography.

The patient was consented to an institutional review board–approved outcomes tracking protocol. After starting proton therapy with image guidance by cone-beam CT, soft tissue differences in the mediastinum were noted at fraction 8, prompting a verification scan and replanning. Clinically, the patient also noted chest pain and some pain with deep breathing. Review of verification cone-beam CT confirmed increased fluid around her heart. Images were reviewed with radiology, confirming recurrent PCE, and her primary medical team was contacted. The patient had a history of anaphylactic reaction to contrast allergy, and for this reason consensus was to evaluate with PET/CT. PET/CT was obtained the next day to evaluate any local and/or distant progression, which lacked FDG avidity in the pericardium and elsewhere, although again confirming new PCE compared with her last PET/CT with a slight increase in mediastinal lymphadenopathy and a Deauville 3 response (**Figure 3**).

**Figure 4.**
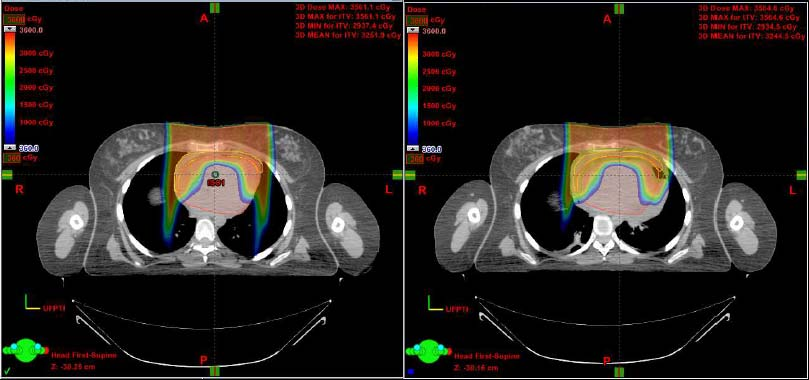
Represented are the scan at the time of simulation (left panel) and the original plan at the time of pericardial effusion recurrence (right panel).

With consensus from her primary team, she was sent to the emergency room for a workup and management of her PCE. Echocardiographic examination noted early tamponade physiology with a stable clinical picture. Given the recurrence, cardiothoracic surgery was recommended and the patient underwent pericardial window formation. Cytology was negative for malignant cells. She was subsequently discharged and resumed proton therapy after replanning, missing just 1 day of treatment. In concordance with her primary team, given the concerns of progression, the ultimate dose prescribed was increased to 41.4 GyRBE. **Figure 4** demonstrates the plan at simulation and subsequent original plan overlaid at time of effusion.

## Discussion

Predicting the occurrence of PCE in patients with neoplasms, such as HL, can prove difficult since PCE may exist subclinically or present unexpectedly. Our case illustrates an uncharacteristically early recurrence of nonmalignant PCE that presented during the beginning of the course of her adjuvant radiotherapy with double-scatter proton therapy. Historically, in HL patients, PCE was reported as one of the earliest known cardiovascular effects of RT, typically as a subacute side-effect with a time course similar to pneumonitis [6]. A review of the modern radiation oncology literature reveals a paucity of information regarding incidence, presentation, and etiology of PCE in adults diagnosed with HL, or reports of patients becoming symptomatic while undergoing RT.

In the United States, acute PCE may occur at some point in 5% of children and up to 24% of adults with HL [7]. Of those diagnosed with PCE, patients may be identified at initial diagnosis of HL, during chemotherapy administration (drug reaction), as a delayed side effect in those who received RT, or as some other preexisting comorbidity, such as kidney failure [8, 9]. Curiously, the Children's Oncology Group found that those with PCE (compared with those without) were more likely to have large mediastinal adenopathy (mediastinal mass that exceeds one third the maximum diameter of the patient's thorax; *P* < .0001), to be older (*P* = .0075), and to be female (*P* = .023) [7, 9].

Large-volume mediastinal lymphadenopathy increases the risk for PCE by obstructing the superior vena cava and physically blocking lymphatic drainage of the pericardium [7]. Patients should be evaluated with electrocardiography, echocardiography (definitive diagnosis), and an X-ray to rule out cardiac tamponade [8]. When PCE is in the presence of HL, F-18 FDG PET/CT holds a high negative predictive value of 94.5% and can be used to help rule out malignant causes [10]. Progression to cardiac tamponade holds a 1-year mortality rate of 79% in the setting of malignancy and 27% in those with PCE attributed to nonmalignant causes. Intervention for moderate-to-large PCE includes therapeutic/diagnostic pericardiocentesis [11]. This proves problematic as pericardiocentesis is an associated risk factor for PCE recurrence [12, 13]. Definitive treatment for PCE includes pericardiectomy or cardiac window [8].

Chemotherapy as first-line treatment for HL typically consists of an anthracycline-containing regimen, such as doxorubicin, bleomycin, vinblastine, and dacarbazine [14]. Clinicians should be aware of the possibility of PCE development during administration of anthracyclines [15]. Treatment includes removal of the offending drug from the chemotherapy regimen, and, if PCE becomes life-threatening (tamponade), intervention with pericardiocentesis may be indicated [16]. Immune checkpoint inhibitors have also been associated with PCE, among other cardiotoxicities [17], which would be of particular importance in the refractory or recurrent setting.

The occurrence of PCE during RT creates a multitude of problems for both the patient and the treating physician outside of the already notable cardiac implications. As for the etiology for our specific patient, it is unclear what led to the recurrence of PCE, and the timeline recognized for radiotherapy-PCE is inconsistent for what our patient experienced. Change in mediastinal anatomy due to volume expansion (as in our case) influences radiation dosimetry and necessitates changes in planning target volume. Patient safety and treatment efficacy may become jeopardized due to possibly missing or underdosing the target or overdosing normal tissues. Patient illness and the need for treatment replanning may result in treatment gaps, delays, or both. In our case, onboard imaging by cone-beam CT allowed for early detection and further investigation, which led to patient referral to surgery. For settings that do not use 3-dimensional image guidance, verification scans may be necessary to ensure appropriate target coverage in addition to any early detection of PCE.

### Conclusion

PCE can be attributed to many causes for survivors of Hodgkin lymphoma, including disease, chemotherapy, immune checkpoint inhibitors, and radiotherapy that are all associated. Acute PCE as a result of radiotherapy is extremely rare with modern treatment techniques, and we present a case of a patient undergoing consolidative proton therapy for Hodgkin lymphoma in which it is unclear what led to recurrence of her PCE. While PCE has been documented as occurring before RT, with the administration of chemotherapeutic drugs or at diagnosis, recurrence is not generally considered within the timeline of when the patient presents for RT planning. It is important for the radiation oncology team to be aware of this potential complication, especially in the setting of a patient with known recurrence, and use on-board imaging alongside patient symptomatology for prompt identification and management.
